# Growing Up Unequal: Disparities of Childhood Overweight and Obesity in Indonesia’s 514 Districts

**DOI:** 10.3390/healthcare11091322

**Published:** 2023-05-05

**Authors:** Wahyu Sulistiadi, Dian Kusuma, Vilda Amir, Dwi Hapsari Tjandrarini, Made Agus Nurjana

**Affiliations:** 1 Department of Health Administration and Policy, Faculty of Public Health, Universitas Indonesia, Depok 16424, Indonesia; 2 Department of Health Services Research and Management, School of Health & Psychological Sciences, City University of London, London EC1V 0HB, UK; 3 Center for Health Administration and Policy Studies, Faculty of Public Health, Universitas Indonesia, Depok 16424, Indonesia; 4 Research Center for Public Health and Nutrition, National Research and Innovation Agency, Bogor 16915, Indonesia

**Keywords:** childhood, overweight, obesity, inequality, geographic, socioeconomic

## Abstract

Background: Childhood obesity is a major public health concern as it increases the risk of premature death and adult disability. Globally, the latest estimates showed that more than 340 million children and adolescents between the ages of 5 and 19 were overweight or obese in 2016. This study aimed to investigate the disparities in childhood overweight and obesity across 514 districts in Indonesia, based on geographic and socioeconomic factors. Methods: Geospatial and quantitative analyses were performed using the latest Indonesian Basic Health Survey data from 2018. Dependent variables were rates of overweight and obesity among children aged 5–17 years including by gender. Results: This study found that the rates of overweight were 17.2%, 17.6%, and 16.8% among all children, boys, and girls, while the rates of obesity were 7.0%, 7.9%, and 6.1%, respectively. Boys were 1.30 times more likely to be obese than girls, while overweight was similar between both sexes. Urban cities had significantly higher prevalence of childhood overweight and obesity compared with rural districts by up to 1.26 and 1.32 times, respectively. In addition, the most developed region had significantly higher prevalence of childhood overweight and obesity than the least developed region by up to 1.37 and 1.38 times, respectively. With regard to socioeconomic factors, our analysis demonstrated a notable disparity in the prevalence of childhood overweight and obesity across income quintiles. Specifically, the wealthiest districts exhibited a 1.18 times higher prevalence of overweight and obesity among all children compared with the poorest districts. This association was particularly pronounced among boys; in the richest quintile, the prevalence of overweight and obesity was 1.24 and 1.26 times higher, respectively, in comparison to the poorest income quintile. In contrast, district-level education appears to exhibit an inverse relationship with the prevalence of childhood overweight and obesity, although the findings were not statistically significant.

## 1. Introduction

Obesity during childhood is linked with increased risk of premature death and adult disability [1]. Children who are overweight or obese are more likely to continue being obese throughout adulthood, and they have a higher risk of developing noncommunicable diseases such as cardiovascular diseases, diabetes, musculoskeletal disorders, and cancers at an earlier age [1]. Globally, the latest estimates showed that more than 340 million children and adolescents between the ages of 5 and 19 were overweight or obese in 2016 [2].

Indonesia, a lower-middle-income country with a population of over 280 million people, is experiencing an increase in childhood overweight and obesity, as observed in many low- and middle-income countries (LMICs) [3]. According to the 2018 Indonesian Basic Health Survey (RISKESDAS), among children aged 5–12 years, the prevalence of overweight and obesity was 20% and 9.2%, respectively [4].

Previous studies have reported evidence on the disparity in prevalence of overweight and obesity among children in relation to socioeconomic status and geographic location [5,6,7,8,9]. A recent literature review conducted in 2021 analyzed 50 papers and discovered that high-income countries showed a positive association between childhood disadvantage (lower family income and parental education) and greater fat mass, while middle-income countries displayed a negative association [5]. In the United Kingdom, a study involving over 21,000 children revealed that children from low-income families were up to three times more likely to be obese compared with those from high-income families [6]. Additionally, a study involving nearly 5000 children in Australia found that the risk of persistent childhood overweight was highest among children from low socioeconomic backgrounds [7]. Among LMICs, a study of over 15,000 children in Bangladesh found that households with a higher wealth index had a higher likelihood of childhood overweight, and higher household education level was also associated with childhood overweight [8]. In China, a study of nearly 10,000 children aged 5–12 years showed that higher per-capita household income and maternal education were associated with an increased risk of overweight and obesity, especially among older boys [9].

It is important to address inequality in childhood obesity in order to achieve Sustainable Development Goal Target 3.4.1, which aims to reduce premature mortality from NCDs [1]. However, the current research on the disparities in childhood overweight and obesity is limited in two main ways. Firstly, the majority of studies have utilized individual-level data, including previous studies from Asia, Europe, and Australia [5,6,7,8,9]. Although these types of analyses are valuable, it is also necessary to examine locality-level data, such as at the district level, particularly in countries including Indonesia where comparatively more decision-making power operates at the district level. Moreover, previous study of RISKESDAS data has lacked geospatial and socioeconomic disparity analysis [4]. Secondly, previous research primarily focused on high-income countries of North America or Europe, or on Australia, with limited studies conducted in LMICs [5,6,7,8,9,10,11,12,13,14]. Consequently, this current study aimed to investigate the disparities in childhood overweight and obesity across 514 districts in Indonesia, based on geographic and socioeconomic factors.

## 2. Methods

### 2.1. Study Design

This is a cross-sectional study on geographic and socioeconomic differences in childhood overweight and obesity rates among 514 districts across 34 provinces in Indonesia. The primary data source was RISKESDAS 2018, which is a nationally representative health survey that collects information on overweight and obesity rates [4].

RISKESDAS surveyed 300,000 households from 30,000 census blocks through a two-stage sampling process. Firstly, the survey team selected 180,000 census blocks out of 720,000 listed in the 2010 population census, using probability proportional to size. Secondly, they picked 30,000 census blocks each from urban and rural areas, again using probability proportional to size. To maintain the variation among households, the team chose ten households according to implicit stratification of the education levels of household heads. The team then interviewed each household member and assessed those who met the inclusion criteria. The response rate to interviews was high, at 95% across the country with a range from 85% in Papua province to 99% in Bangka Belitung province. The survey included a total sample of 1,017,290 persons interviewed. For this study, a total sample of 265,469 children 5–17 years old (48.2% girls and 51.8% boys) were included [4].

### 2.2. Independent Variables

The study analyzed four independent variables at the district level, namely region, urbanicity, income, and education. The data for these variables were from the World Bank database [15]. The provinces and districts were classified into five regions: Sumatera, Java and Bali, Kalimantan, Sulawesi, and Papua/Nusa Tenggara/Maluku. The western regions (e.g., Java and Bali) are economically more developed than the eastern regions (e.g., Papua, Nusa Tenggara, and Maluku) [16,17,18]. The analysis was conducted for all 514 districts, 97 cities (urban districts), and 417 regencies (rural districts).

The study used poverty rates as a proxy for district-level income level. Poverty rates of districts were divided into five groups, with the poorest in the first quintile (highest poverty rates) and the wealthiest in the fifth quintile (lowest poverty rates). To measure poverty, the Indonesia Statistics Bureau (BPS) uses the concept of the basic needs approach, referring to the *Handbook on Poverty and Inequality* published by the World Bank. With this approach, poverty is seen as the inability, from an economic standpoint, to meet basic food and non-food needs that are measured in terms of expenditure. Individuals are categorized as poor if their average per capita monthly expenditure is below the poverty line [19]. Similarly, the net enrollment ratios for senior secondary education were categorized into five groups, with the least educated in the first quintile and the most educated in the fifth quintile [20,21]. The reference map is available in Appendix A.

### 2.3. Dependent Variables

This study examined two indicators of childhood obesity and overweight as the outcome variables, and these two indicators were examined in groups stratified by sex/gender. The indicators were overweight among children, boys, and girls, and obesity among children, boys, and girls. The WHO’s criteria for defining overweight and obesity in children aged 5–19 years were used. Overweight is when the BMI-for-age is more than 1 standard deviation above the median of the WHO Growth Reference, and obesity is when the BMI-for-age is more than 2 standard deviations above the median of the WHO Growth Reference [2]. The indicators by sex including for boys and girls were considered to assess potential sex-based variations, which could help improve NCD control and prevention efforts and health-system reforms in Indonesia and other LMICs [22].

### 2.4. Statistical Analysis

This study employed both geospatial analysis and multivariate regression to analyze data from 514 districts across 34 provinces. The provincial and district-level obesity data were divided by quintile using ArcMap 10. We analyzed the associations between exposure indicators, such as region, urbanicity, income, and education, and each of the six overweight/obesity outcome indicators. This study calculated the absolute and relative differences for geographic and socioeconomic variations, and compared the differences between the most and least developed regions and between the poorest and wealthiest/most educated quintiles. To compare the differences, ordinary least square (OLS) regressions were employed in STATA 15. Statistical significance was set at the 5% level or lower.

## 3. Results

### 3.1. Provincial Level Results

**Figure 1:**
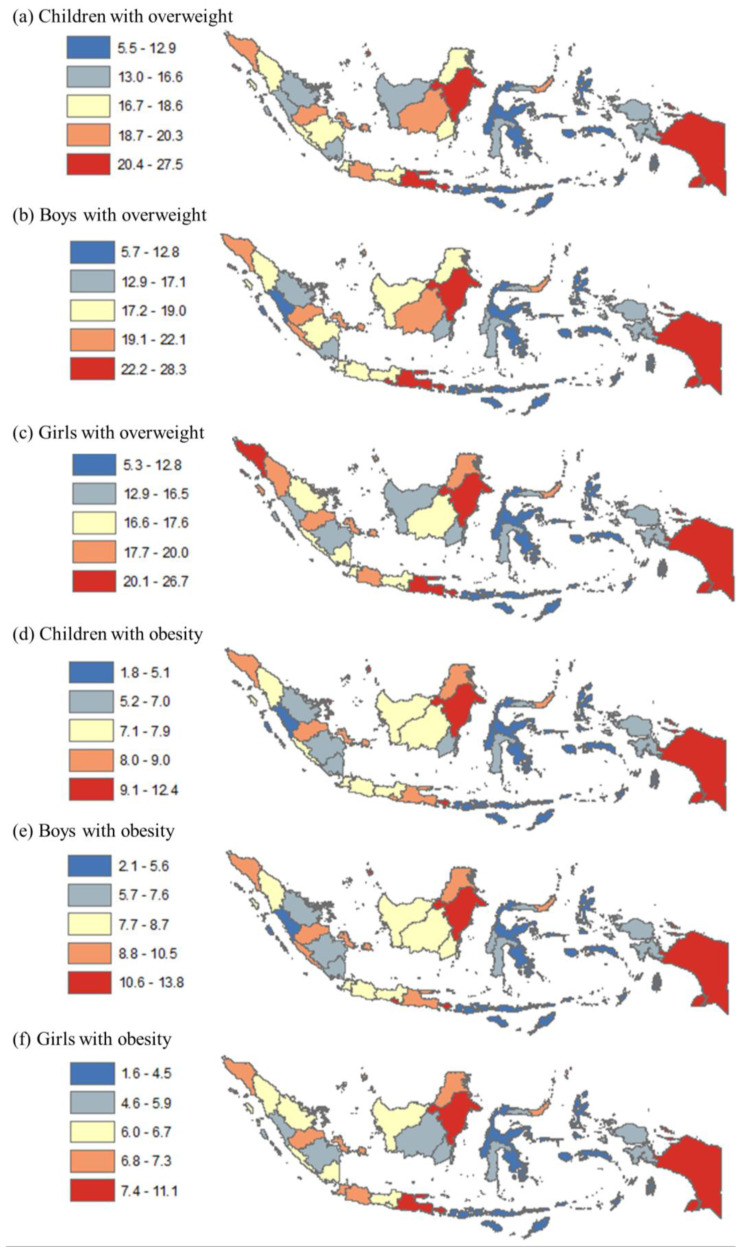
Disparity in rates of childhood overweight and obesity by province in Indonesia, 2018. Note: Numbers show prevalence of overweight and obesity among all children, boys, and girls. The map was created by the authors in ArcMap 10.

In Figure 1, the prevalence of childhood overweight and obesity is shown by quintile at the province level. Panels a–c show the range of overweight prevalence among all children, boys, and girls, which varied from 5.5% to 27.5%, 5.7% to 28.3%, and 5.3% to 26.7%, respectively. Panels d–f show the range of obesity prevalence among all children, boys, and girls, which varied from 1.8% to 12.4%, 2.1% to 13.8%, and 1.6% to 11.1%, respectively. High overweight prevalence among all children was found in many provinces in Java and Bali, Sumatera, Kalimantan, and some provinces in other regions (e.g., Sulawesi and Papua). Similarly, the highest obesity prevalence among all children was in many provinces in Java, Sumatera, Kalimantan, and two provinces in other regions. The pattern for both childhood overweight and obesity was relatively similar by sex.

Table 1 displays the frequency of childhood obesity and overweight for each province. The top and bottom boxes highlight the ten provinces with the lowest and highest poverty rates, respectively. The shaded cells signify that the prevalence in that province is higher than the national average (bottom row) for each outcome indicator (columns 2–7). Eight of the wealthiest provinces had higher than average prevalence for at least five categories, whereas only three of the poorest provinces had the same.

### 3.2. District Level Results

Table 2 provides statistics for the districts in this study, including the prevalence of childhood overweight and obesity. This study analyzed all 514 districts, with 97 (18.9%) classified as urban cities and 417 (81.1%) as rural regencies. Urban cities were predominantly located in Java (36.1% of 97) and Sumatera (34.0%), while rural regencies were more dispersed, in Java (29.0%), Sumatera (22.3%), Papua (20.6%), Sulawesi (16.8%), and Kalimantan (11.3%). According to income level, 79% of urban areas were in quintiles 4–5 (wealthier), while almost half (47.2%) of rural areas were in quintiles 1–2 (poorer). In terms of education level, 71.1% of urban cities were in quintiles 4–5 (higher education), while almost half (46.8%) of rural regencies were in quintiles 1–2 (lower education). In terms of the outcome variables, the prevalence of overweight was 17.2%, 17.6%, and 16.8%, while that of obesity was 7.0%, 7.9%, and 6.1% among all children, boys, and girls, respectively. Our analysis revealed that childhood overweight and obesity were significantly more prevalent in urban areas than rural areas (see Appendix B for the statistical tests). Among all children, boys, and girls, overweight rates were 20.4%, 21.1%, and 19.8% in urban areas and 16.5%, 16.8%, and 16.2% in rural areas. Obesity rates were 8.6%, 9.9%, and 7.3% in urban areas and 6.7%, 7.5%, and 5.8% in rural areas.

**Figure 2:**
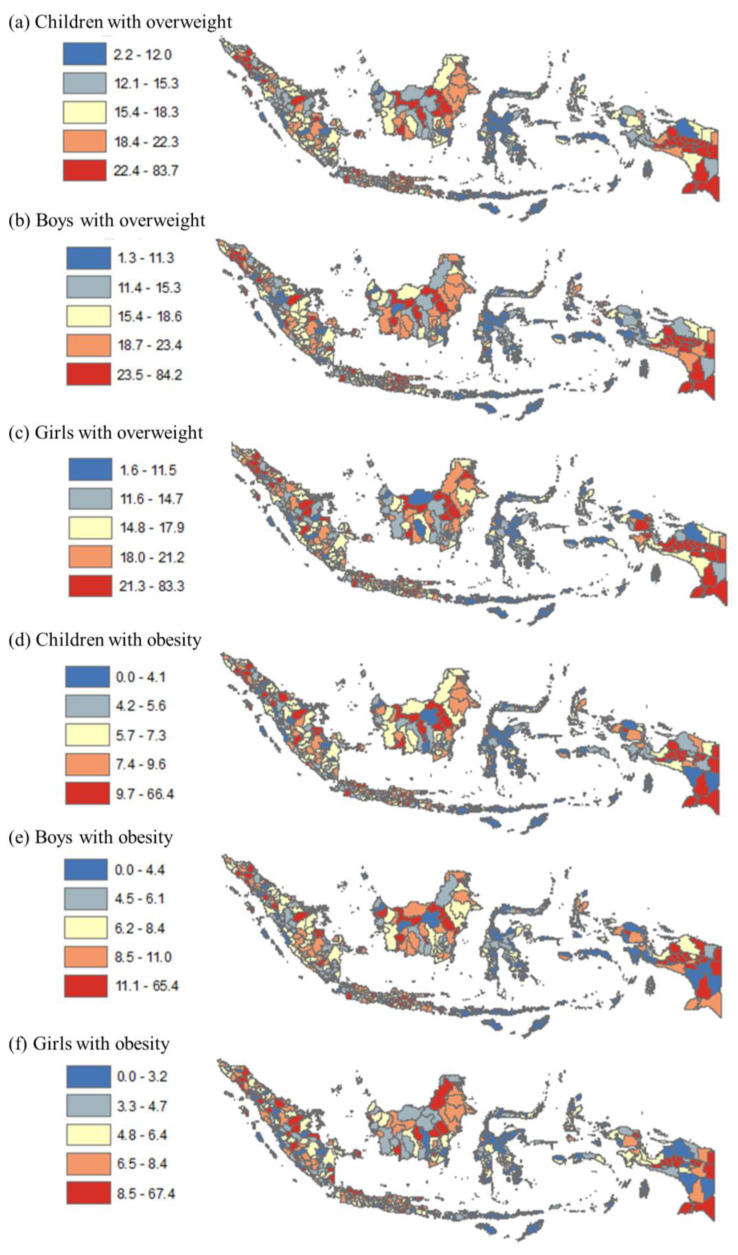
Disparity in prevalence of childhood overweight and obesity by district in Indonesia, 2018. Note: Numbers show prevalence of overweight and obesity among all children, boys, and girls. The map was created by the authors in ArcMap 10.

Figure 2 provides a more detailed view of childhood overweight and obesity prevalence by quintile at the district level compared to the provincial level. The figure indicates that many districts in Riau, South Sumatera, Central Java, West Kalimantan, and West Papua provinces had the highest prevalence among all children for both overweight and obesity (quintiles 4–5).

Table 3 and Table 4 display the associations between childhood overweight/obesity, geographic region, and the socioeconomic factors of income and education. The absolute and relative values show the disparity between the most and least developed areas, the wealthiest and poorest districts, and the most and least educated districts. More developed regions had a higher occurrence of childhood overweight and obesity compared with less developed regions. Specifically, districts in Java had 32% higher prevalence of overweight among all children, 37% higher among boys and 26% higher among girls, compared with those in Papua. Similarly, they had 32% higher prevalence of obesity among all children, 38% higher among boys and 27% higher among girls, compared with those in Papua. In terms of socioeconomic factors, the wealthiest districts had higher prevalence of childhood overweight and obesity compared with the poorest districts—these results were statistically significant in the bivariate analyses but not in the multivariate analyses. The districts in the richest income quintile had 18% higher prevalence of overweight among all children, 24% higher among boys and 10% higher among girls, compared with those in the poorest income quintile. Similarly, they had 18% higher prevalence of obesity among all children, 26% higher among boys and 9% higher among girls, compared with those in the poorest income quintile. In contrast, the association between childhood overweight and obesity with education was generally negative (i.e., higher levels of education are correlated with lower prevalence of overweight/obesity among children) (although the findings were not statistically significant).

## 4. Discussion

This current study revealed a substantial prevalence of overweight and obesity in children aged 5–17 years in Indonesia. According to the WHO definition, the prevalence of overweight was 17.2%, 17.6%, and 16.8% among all children, boys, and girls, while the prevalence of obesity was 7.0%, 7.9%, and 6.1%, respectively. These findings place Indonesia in Stage 1 of the obesity transition, which is a more advanced stage compared with other Southeast Asian countries with higher per capita gross national income, such as the Philippines and Thailand [23]. In the Philippines, the prevalence of overweight among boys and girls was 9.2% and 8.6%, and the prevalence of obesity was 4.3% and 3.1% [24]. In Thailand, the prevalence of overweight among boys and girls was 22% and 16.1%, and the prevalence of obesity was 8.7% and 4.7%, respectively [24].

Our study revealed substantial disparities in childhood overweight/obesity across 514 districts in Indonesia, based on geographic and socioeconomic factors. Urban districts (i.e., cities) had significantly higher prevalence of overweight and obesity among all children, boys, and girls than rural districts (i.e., regencies). Similar findings have been reported in previous studies conducted in other LMICs. For instance, a study in Bangladesh found a higher prevalence of overweight among urban children, particularly boys, compared with rural children [8]. Similarly, a study using data from the Chinese national survey on students’ health showed that overweight and obesity were more common in urban areas [25]. This study also found that the more developed regions (Java and Bali) had higher prevalence of childhood overweight and obesity compared with the less developed regions (Papua, Nusa Tenggara, and Maluku). This may be due to the higher number of urban districts in developed regions—70.1% of urban districts (cities) are in the Java/Bali and Sumatera regions. The urban–rural obesity differential is due to a range of factors, including shifts in infrastructure, transportation, employment, income, food availability, and levels of physical activity. These factors tend to intensify in economically developed and urbanized regions, leading to a greater prevalence of obesity including among children [26].

With regard to socioeconomic factors, our analysis demonstrated a notable disparity in the prevalence of childhood overweight and obesity across income quintiles. Specifically, the wealthiest districts exhibited a 1.18 times higher prevalence of overweight and obesity among all children compared with the poorest districts. This association was particularly pronounced among boys; in the richest quintile, the prevalence of overweight and obesity was 1.24 and 1.26 times higher, respectively, in comparison with the poorest income quintile. Conversely, higher district-level education appeared to be correlated with a reduced prevalence of childhood overweight and obesity, although the findings were not statistically significant. In the literature, the associations between childhood overweight/obesity and levels of income and education are complex and multifaceted. In some LMICs, children from higher-income families and those with higher levels of education are more likely to be overweight or obese. This is often due to a shift in dietary patterns towards more calorie-dense foods and less physical activity as countries undergo economic development and urbanization [26,27]. However, in many LMICs, children from low-income families are at greater risk of malnutrition and undernutrition including stunting and wasting. In these settings, childhood obesity may be less prevalent overall but can still coexist with undernutrition due to the consumption of energy-dense but nutrient-poor foods [25,27]. Additionally, cultural and environmental factors play significant roles in shaping dietary and physical activity behaviors in LMICs, which can vary widely by region, country, and socioeconomic group [5,8,28].

Regarding policy, it is noted that childhood overweight and obesity rates are high in Indonesia, comparable to the rates in other countries with higher economic development such as Thailand, an upper-middle income country. This suggests that effective interventions and strategies to reduce childhood obesity and its geographic/socioeconomic disparities are critical [26,28]. This is particularly important because children who are overweight or obese are more likely to continue being obese into adulthood, leading to lower productivity and higher health costs due to obesity-related illnesses among the working population [29]. The prevalence of childhood overweight and obesity emphasizes the requirement to redirect the healthcare system’s focus on preventing and managing obesity and other risk factors across all levels of care, starting from community care and primary to secondary and tertiary services, possibly through integration with infectious disease programs [30,31,32,33,34,35]. Overall, the complexity of relationships between childhood obesity, income, and education levels in LMICs highlights the need for context-specific interventions that address the underlying factors contributing to obesity risk, taking into account cultural, social, and economic factors [3,27].

While this current study is the first of its kind to utilize a significant number of subnational units in an LMIC, it has at least two limitations. Firstly, the data lacked an ethnicity variable, which restricted our ability to conduct ethnicity-based sub-group analysis [34]. Secondly, the cross-sectional design prevented us from assessing trends in childhood overweight and obesity over time. Despite these limitations, our findings are significant for policy, particularly for preventing and controlling non-communicable diseases among children in LMICs.

## 5. Conclusions

Our study discovered that the prevalence of overweight among children was 17.2% overall, with 17.6% among boys and 16.8% among girls. Meanwhile, the rates of obesity were 7.0% overall, with 7.9% among boys and 6.1% among girls. Boys had a 1.30 times higher likelihood of obesity compared with girls, while overweight was similar among both genders. Urban cities showed significantly higher prevalence of childhood obesity and overweight compared with rural areas by 1.26 and 1.32 times, respectively. Furthermore, the most developed region had significantly higher prevalence of childhood obesity and overweight than the least developed region by 1.37 and 1.38 times, respectively. Our study highlights the notable influence of socioeconomic factors, particularly income, on the prevalence of childhood overweight and obesity. Districts with higher income levels display a greater prevalence of these health issues, with boys being disproportionately affected. On the other hand, district-level education appears to have an inverse relationship with the prevalence of childhood overweight and obesity, although the findings were not statistically significant. Ultimately, our findings underscore the importance of developing targeted interventions that address disparities to effectively combat the growing issue of childhood overweight and obesity.

## Figures and Tables

**Table 1:** Prevalence of childhood overweight and obesity by province.

	Poverty	Overweight (%)			Obesity (%)		
	Rates (%)	All	Boys	Girls	All	Boys	Girls
	(1)	(2)	(3)	(4)	(5)	(6)	(7)
Bali	4.5	22.3	24.2	20.3	9.6	11.5	7.5
South Kalimantan	4.8	16.8	17.1	16.5	7.0	8.1	5.8
Central Kalimantan	5.0	18.8	20.4	17.1	7.3	8.6	5.9
Jakarta	5.0	27.4	28.3	26.5	12.4	13.8	10.9
Banten	5.3	17.5	17.8	17.1	7.9	8.4	7.3
Bangka Belitung	5.4	19.4	19.5	19.3	8.4	9.4	7.3
West Sumatera	6.6	13.6	12.7	14.5	5.1	5.2	5.0
North Kalimantan	7.0	18.6	18.2	18.9	8.2	9.2	7.2
East Kalimantan	7.1	22.3	23.7	20.9	9.8	11.5	8.0
Riau Islands	7.6	20.7	22.1	19.2	9.5	11.9	7.0
Jambi	7.8	19.6	21.0	18.1	8.4	10.0	6.8
North Maluku	7.9	12.5	12.8	12.2	5.1	5.6	4.5
West Java	7.9	19.4	18.9	20.0	7.8	8.3	7.2
West Kalimantan	8.1	16.5	17.5	15.5	7.1	8.1	6.0
North Sulawesi	8.5	20.3	21.0	19.5	8.1	9.0	7.1
Riau	8.8	16.6	16.2	16.9	7.0	7.3	6.7
South Sulawesi	9.8	13.8	14.1	13.4	5.4	6.3	4.6
West Sulawesi	10.3	12.9	13.0	12.8	4.6	5.7	3.4
East Java	10.9	21.9	23.2	20.7	9.0	10.5	7.4
Central Java	10.9	17.4	18.0	16.7	7.2	8.2	6.0
North Sumatera	11.3	18.6	19.0	18.2	7.4	8.7	6.0
Lampung	12.6	16.5	15.8	17.2	6.3	6.1	6.6
Yogyakarta	12.7	20.0	22.3	17.5	9.0	11.5	6.4
Southeast Sulawesi	13.0	11.3	11.8	10.8	4.3	5.2	3.3
South Sumatera	13.1	16.7	17.4	16.0	6.7	7.6	5.9
Central Sulawesi	14.6	10.7	10.6	10.9	3.5	4.1	3.0
West Nusa Tenggara	14.8	9.0	9.2	8.9	3.1	3.5	2.8
Bengkulu	15.0	18.6	19.4	17.6	7.8	8.9	6.7
Aceh	16.4	19.6	19.1	20.1	8.2	9.1	7.3
Gorontalo	16.8	14.7	14.3	15.1	5.4	6.0	4.8
Maluku	21.8	9.6	9.5	9.8	3.7	4.1	3.2
East Nusa Tenggara	22.0	5.5	5.7	5.3	1.8	2.1	1.6
West Papua	26.5	15.5	14.7	16.3	5.8	6.5	5.1
Papua	29.4	27.5	28.3	26.7	12.4	13.6	11.1
AVERAGE		17.1	17.6	16.7	7.1	8.1	6.0

Note: The provinces are ordered by the average poverty rates in column 1. The provinces in the top box are the ten richest provinces and those in the bottom box are the ten poorest provinces. Shaded values show values higher than the national average for each group.

**Table 2:** Descriptive statistics of districts.

			All		Urban		Rural		Difference
			n	%	n	%	n	%	%
			(1)	(2)	(3)	(4)	(5)	(6)	(7) = (4−6)
(a) Characteristics (#)									
	Sample size district		514	100%	97	100%	417	100%	0%
	Region								
		Papua	95	18.5%	9	9.3%	86	20.6%	−11.3%
		Java	128	24.9%	35	36.1%	93	22.3%	13.8%
		Sumatera	154	30.0%	33	34.0%	121	29.0%	5.0%
		Kalimantan	56	10.9%	9	9.3%	47	11.3%	−2.0%
		Sulawesi	81	15.8%	11	11.3%	70	16.8%	−5.4%
			514		97		417		
	Income								
		Q1 poor	102	19.8%	3	3.1%	99	23.7%	−20.6%
		Q2	103	20.0%	5	5.2%	98	23.5%	−18.3%
		Q3	103	20.0%	13	13.4%	90	21.6%	−8.2%
		Q4	103	20.0%	22	22.7%	81	19.4%	3.3%
		Q5 rich	103	20.0%	54	55.7%	49	11.8%	43.9%
			514		97		417		
	Education								
		Q1 least	103	20.0%	0	0.0%	103	24.7%	−24.7%
		Q2	103	20.0%	11	11.3%	92	22.1%	−10.7%
		Q3	103	20.0%	17	17.5%	86	20.6%	−3.1%
		Q4	103	20.0%	29	29.9%	74	17.7%	12.2%
		Q5 most	102	19.8%	40	41.2%	62	14.9%	26.4%
			514		97		417		
(b) Outcome variables (%)									
	Overweight all		n/a	17.2%	n/a	20.4%	n/a	16.5%	3.9% *
	Overweight boys		n/a	17.6%	n/a	21.1%	n/a	16.8%	4.3% *
	Overweight girls		n/a	16.8%	n/a	19.8%	n/a	16.2%	3.6% *
	Obesity all		n/a	7.0%	n/a	8.6%	n/a	6.7%	1.9% *
	Obesity boys		n/a	7.9%	n/a	9.9%	n/a	7.5%	2.4% *
	Obesity girls		n/a	6.1%	n/a	7.3%	n/a	5.8%	1.5% *

Note: Q = quintile, n = number, % = proportion of column total, Urban = city, Rural = regency. Data on district characteristics are from the World Bank and overweight/obesity data are from Basic Health Survey 2018. Asterisks (*) show statistical significance at 5% level (see Appendix B for the OLS regression outputs conducted in STATA 15).

**Table 3:** Prevalence of childhood overweight and obesity by region, income, and education in Indonesia, 2018.

	N = 514	Overweight			Obesity		
	Districts	All	Boys	Girls	All	Boys	Girls
Region	Papua	15.1%	15.0%	15.2%	6.3%	6.8%	5.6%
	Sulawesi	13.3%	13.6%	13.0%	5.0%	5.6%	4.3%
	Kalimantan	18.6%	19.5%	17.6%	7.9%	9.2%	6.5%
	Sumatera	17.9%	18.1%	17.6%	7.2%	8.2%	6.2%
	Java	19.9%	20.6%	19.2%	8.3%	9.4%	7.1%
	Absolute	**4.8%**	**5.6%**	**4.0%**	**2.0%**	**2.6%**	**1.5%**
	Relative	**1.32**	**1.37**	**1.26**	**1.32**	**1.38**	**1.27**
Income	Q1 poor	17.0%	16.8%	17.2%	7.1%	7.7%	6.4%
	Q2	14.6%	14.8%	14.4%	5.7%	6.5%	5.0%
	Q3	17.5%	18.1%	16.8%	6.9%	8.0%	5.8%
	Q4	17.1%	17.4%	16.8%	6.9%	7.7%	6.1%
	Q5 rich	20.0%	20.9%	19.0%	8.4%	9.7%	7.0%
	Absolute	3.0%	4.1%	1.8%	1.3%	2.0%	0.6%
	Relative	1.18	1.24	1.10	1.18	1.26	1.09
Education	Q1 least	17.4%	17.7%	17.0%	7.2%	7.9%	6.4%
	Q2	15.7%	15.6%	15.7%	6.4%	7.1%	5.7%
	Q3	17.2%	17.7%	16.7%	7.0%	7.9%	6.0%
	Q4	17.5%	18.0%	17.0%	7.2%	8.2%	6.1%
	Q5 most	18.3%	19.0%	17.7%	7.4%	8.5%	6.1%
	Absolute	0.9%	1.3%	0.7%	0.2%	0.6%	−0.3%
	Relative	1.05	1.07	1.04	1.03	1.08	0.95

Note: Q = quintile; Java region includes Bali; Papua region includes Maluku and Nusa Tenggara. Income quintile according to district-level poverty rate (e.g., Q1 = 20% of districts with highest poverty rate). Absolute (relative) = difference (ratio) between Papua and Java, Q1 and Q5. For education, absolute (relative) between Q1 and Q5. Appendix C shows the educational and poverty level by region. Boldface values show statistical significance at 5% level (see Table 4 for the OLS regression outputs conducted in STATA 15).

**Table 4:** Regression outputs for geographic and socioeconomic disparity in childhood overweight and obesity in Indonesia, 2018.

		**Overweight**			**Obesity**		
	**N = 514**	**All**	**Boys**	**Girls**	**All**	**Boys**	**Girls**
	**Districts**	**Coef**	**Coef**	**Coef**	**Coef**	**Coef**	**Coef**
(a) Bivariate analysis							
Region	Papua	Reference					
	Java	4.77 *	5.60 *	3.96 *	2.04 *	2.62 *	1.47 *
	Sumatera	2.73 *	3.13 *	2.40 *	0.96	1.35 *	0.60
	Kalimantan	3.43 *	4.46 *	2.41	1.60 *	2.36 *	0.84
	Sulawesi	−1.84	−1.41	−2.22 *	−1.29	−1.17	−1.37 *
Income	Q1 poor	Reference					
	Q2	−2.42 *	−1.98	−2.81 *	−1.38 *	−1.28	−1.43 *
	Q3	0.46	1.27	−0.32	−0.19	0.27	−0.64
	Q4	0.12	0.64	−0.37	−0.20	−0.04	−0.35
	Q5 rich	2.95 *	4.08 *	1.85	1.27 *	1.97 *	0.58
Education	Q1 least	Reference					
	Q2	−1.76	−2.13	−1.30	−0.78	−0.80	−0.74
	Q3	−0.23	−0.08	−0.34	−0.26	−0.03	−0.47
	Q4	0.10	0.27	−0.02	−0.05	0.26	−0.33
	Q5 most	0.91	1.25	0.62	0.14	0.62	−0.32
(b) Multivariate analysis							
Region	Papua	Reference					
	Java	5.17 *	5.61 *	4.75 *	2.40 *	2.79 *	2.01 *
	Sumatera	3.37 *	3.42 *	3.38 *	1.43 *	1.64 *	1.25
	Kalimantan	2.95 *	3.46 *	2.43	1.49	2.04 *	0.92
	Sulawesi	−0.67	−0.44	−0.87	−0.64	−0.58	−0.68
Income	Q1 poor	Reference					
	Q2	−2.85 *	−2.44 *	−3.23 *	−1.49 *	−1.50 *	−1.44 *
	Q3	−1.55	−0.89	−2.19	−1.10	−0.87	−1.32
	Q4	−1.53	−1.13	−1.91	−0.93	−0.99	−0.86
	Q5 rich	1.00	1.89	0.12	0.35	0.71	−0.00
Education	Q1 least	Reference					
	Q2	−1.76	−2.33 *	−1.11	−0.67	−0.74	−0.59
	Q3	−0.23	−0.24	−0.19	−0.14	0.03	−0.32
	Q4	−0.45	−0.42	−0.45	−0.21	0.04	−0.45
	Q5 most	−0.35	−0.21	−0.47	−0.33	0.04	−0.72

Note: Coef = coefficients, Q = quintile; Java region includes Bali; Papua region includes Maluku and Nusa Tenggara. Income quintile according to district-level poverty rate (e.g., Q1 = 20% of districts with highest poverty rate). Absolute (relative) = difference (ratio) between Papua and Java, Q1 and Q5. For education, absolute (relative) between Q1 and Q5. Bivariate and multivariate analyses were conducted using OLS regressions in STATA 15. Asterisk (*) indicates statistical significance at 5% level or lower.

**Figure A1:**
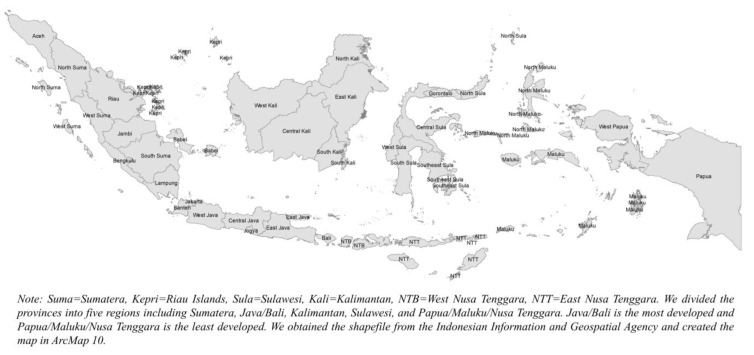
Map of provinces in Indonesia.

## Data Availability

Available from the corresponding author upon reasonable request.

## Appendix B

**Table A1:** Regression outputs for urban/rural differences.

	Overweight All	Overweight Boys	Overweight Girls	Obesity All	Obesity Boys	Obesity Girls
	Coef	Coef	Coef	Coef	Coef	Coef
						
Rural	Reference					
Urban	3.94 **	4.32 **	3.60 **	1.96 **	2.47 **	1.48 **
						
Constant	16.49 **	16.78 **	16.15 **	6.65 **	7.47 **	5.78 **
						
Observations	514	514	514	514	514	514
R-squared	0.04	0.04	0.03	0.03	0.04	0.02

Note: Coef = OLS Coefficient; Significance level ** *p* < 0.01.

## Appendix C

**Table A2:** Educational and poverty level by region.

Region	Education Enrolment Ratio (%)	Poverty Rates (%)
Papua	53.8	22.6
Sulawesi	61.4	11.6
Kalimantan	57.2	6.3
Sumatera	66.2	11.2
Java	62.6	9.3
Total	61.3	12.3
